# Inter-cellular adhesion disruption and the RAS/RAF and beta-catenin signalling in lung cancer progression

**DOI:** 10.1186/1475-2867-8-7

**Published:** 2008-05-20

**Authors:** Rudolf Götz

**Affiliations:** 1 Institut für Medizinische Strahlenkunde und Zellforschung (MSZ), Universität Würzburg, Versbacher Straße 5, 97078 Würzburg, Germany

## Abstract

Cadherin cell adhesion molecules play an essential role in creating tight intercellular association and their loss has been correlated with poor prognosis in human cancer. Mutational activation of protein kinases and loss of cell adhesion occur together in human lung adenocarcinoma but how these two pathways interconnect is only poorly understood. Mouse models of human lung adenocarcinoma with oncogene expression targeted to subtypes of lung epithelial cells led to formation of adenomas or adenocarcinomas that lacked metastatic potential. Conditional genetic abrogation of epithelial tumour cell adhesion in mice with benign lung tumours induced by oncogenic RAF kinase has been demonstrated to induce intratumourous vascularization (angiogenic switch), progression to invasive adenocarcinoma and micrometastasis. Importantly, breaking cell adhesion in benign oncogene-driven lung tumour cells activated β-catenin signalling and induced the expression of several genes that are normally expressed in intestine rather than the lung. I will discuss potential routes to nuclear β-catenin signalling in cancer and how nuclear β-catenin may epigenetically alter the plasticity of tumour cells during malignant progression.

## Background

Lung cancer has become the most prevalent neoplasm throughout the world with 1,2 million deaths per year [1]. Non-small-cell lung cancer (NSCLC), with its main subtypes adenocarcinoma, squamous cell and large-cell carcinoma is the most frequent type (~80%) of lung cancer with high metastatic potential and low cure rate. As is true for most types of cancer, clinical onset of disease is noted typically in people at an age over-fifties [2]. Since the incidence of lung cancer was very low only five generations ago, it is clear that changes in life style have caused the surge of this disease [3]. Cigarette smoking is a major cause of lung cancer, but about 25% of lung cancer cases are found in non-smokers [4] and these may be attributable to passive smoking or other environmental causes. Quite often, the primary lung tumour is inoperable at the time of diagnosis because regional lymphatic and distant organ metastases are already present [5]. Thus, progressed disease state may in part explain the high mortality rates. The knowledge of the cellular and genetic origin of lung cancer is incomplete, because its detection in early stages using computer tomography scans is only in its beginnings [6]. Significant progress has been made in identifying genetic lesions in lung cancer by sequencing-based mutation screening [7] and by characterizing copy-number alterations [8]. Mouse models of the human disease offer the opportunity that the effects of oncogenes or other genetic and epigenetic factors thought to underlie the hallmarks of lung cancer can be studied and stages of disease progression can presumably be identified [9]. Furthermore, mouse modelling of human lung cancer provides a potentially powerful system for evaluation of novel therapeutic agents that may be clinically useful. Finally, such mice will be valuable tools to identify the origin of the cell(s) from which a lung tumour arises, the cancer stem cell or tumour initiating cell [10,11].

### Target cells for oncogenic transformation in the distal mouse lung

**Figure 1:**
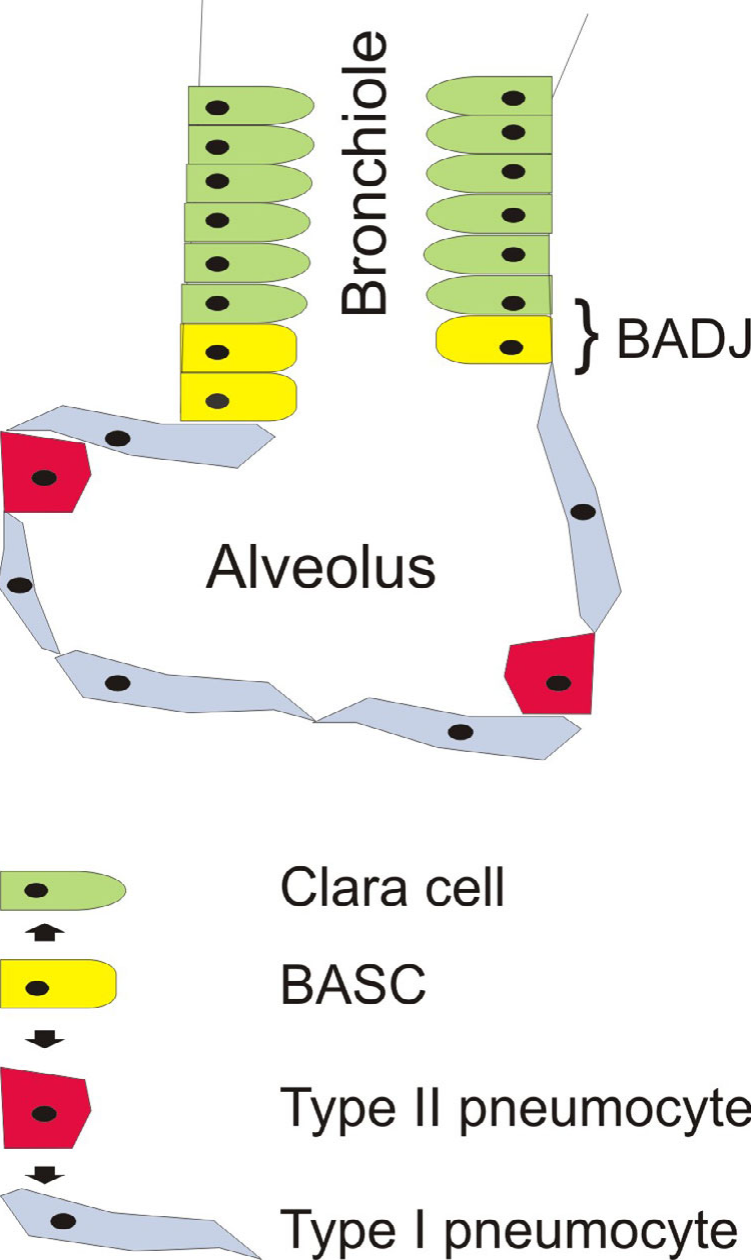
**Epithelial cell types in the distal mouse lung**. Epithelial cell types present in the distal lung that might be target cells for oncogenic transformation are the bronchio-alveolar stem cell (BASC) that localizes to the   bronchio-alveolar duct junction (BADJ) and more differentiated cells with specific functions such as type II pneumocytes and Clara cells. The cuboidal alveolar type II cells as well as the columnar Clara cell may assume progenitor cell status in case of injury. Whether terminally differentiated type I pneumocytes can be recruited into tumours is not known.

The major function of the lung is gas exchange. The epithelial cells which line the tree of tubules and alveolar units arise from progenitor cells present in the lung bud of the ventral foregut endoderm. During embryonic development, they form the epithelial lining of the conducting airways and the distal vascularized sac-like structures. At birth, fluid is resorbed from these sacs to expose a functional gas exchange surface. The subsequent postnatal maturation of the lung involves the formation of septa in the terminal sacs (alveologenesis) leading to an expansion of the gas exchange surface [12]. Thus, in the adult lung distinct types of epithelial cells line the trachea, bronchi, bronchioli and alveoli. The alveolar regions of the human lung make up a surface of roughly 100 m^2^, whereas the conducting airways cover less than 1% of this surface. Early studies [13] on the nuclear incorporation of radio labelled nucleotides and the maintenance of the label-retaining cells in pulse-chase experiments showed that epithelial cells of the mature rodent lung have a half-life of about 100 days and that there could exist distinct types of label-retaining cells – that may be stem cells – in different anatomical regions of the adult lung. In line with this notion, in case of injury or cell death caused by infections, quiescent cells start to proliferate and replace the lost cells. The topic of lung epithelial stem cells has been reviewed recently elsewhere [14,15]. Alveolar type II pneumocytes secrete a variety of pulmonary surfactant proteins (SP), for example SP-C [16]. Type II cells are also the precursors of type I cells which form the thin diffusion barrier important for gas exchange in the alveolus (Figure 1). In mice, SP-C expression is not restricted to type II cells, since rare cells located at the bronchio-alveolar duct junction (BADJ, Figure 1) co-express SP-C together with the Clara cell antigen CC10, a marker specific for Clara cells, another epithelial cell that normally lines the bronchi and bronchioles [17]. The double-positive cell pool has been shown to be resistant to naphthalene treatment which destroys all other Clara cells lining the bronchus and the terminal bronchioles. Furthermore, these cells have been isolated based on their Sca1/CD34 phenotype and been shown to be capable of self-renewal and differentiation in vitro and thus have been called bronchio-alveolar stem cells (BASCs, [18]). BASCs may however be only one of several cell types that have stem cell properties and can repair injured lung. Type II cells have also been shown to re-enter the cell cycle and proliferate in radiation-induced alveolar injury [19] and in bleomycin-induced destruction of type I and type II alveolar cells [18,20]. During repair of alveolar damage, type II cells differentiate into type I cells. It is notable, that type II alveolar cells proliferate in idiopathic pulmonary fibrosis, a proliferative lung disease [21].

### Mutations in NSCLC and their corresponding mouse models

**Figure 2:**
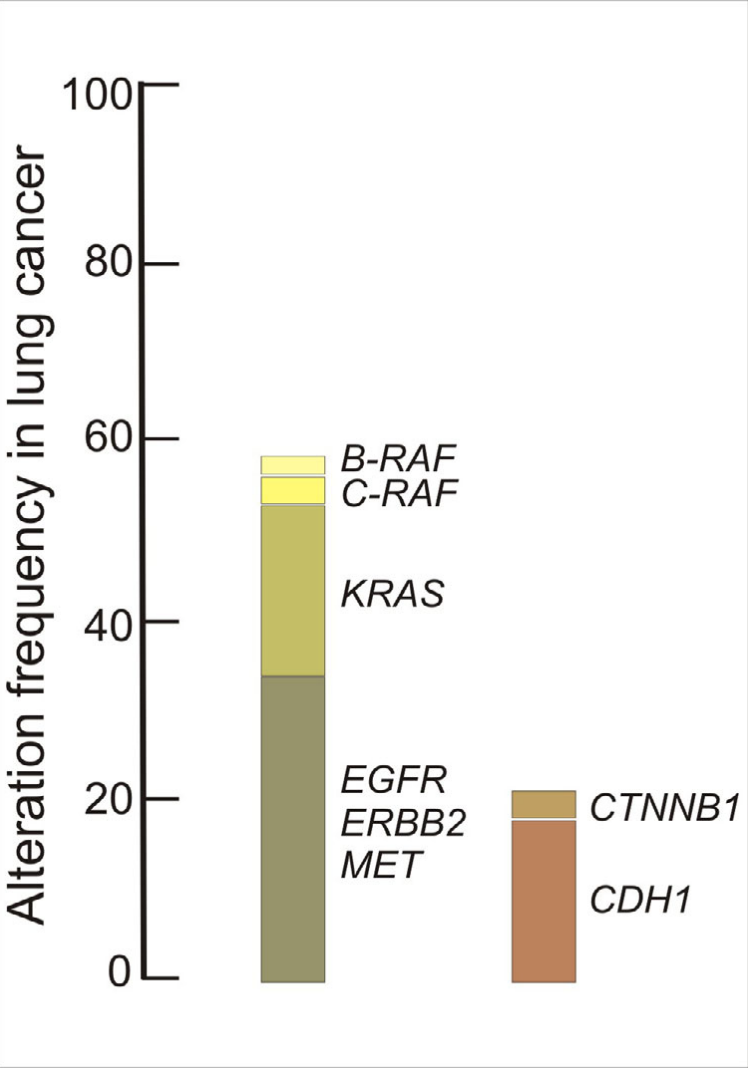
**Mutation frequency in NSCLC**. Frequency of activating point mutations and copy number amplifications found in NSCLC in genes specifying components of the classical mitogen activated protein kinase (MAPK) signaling cascade. The majority of alterations are point mutations that convert the proto-oncogene into a constitutive active oncogenic form. Frequencies for mutation-activated β-catenin (CTNNB1) and silencing of the promoter region of *CDH1 *encoding E-Cadherin are shown. Data are derived from literature and the COSMIC database (version 35).

I will focus here on genes specifying components of the classical mitogen activated protein kinase (MAPK) signalling cascade which are among those most frequently overexpressed, amplified or mutated in NSCLC (Figure 2). Starting at the cell surface, overexpression of members of the ErbB receptor family, most notably the epidermal growth factor receptor (EGFR), and of some of its ligands, such as EGF and TGF-α, has been observed [8,22]. In addition, mutations have been detected in NSCLC samples in the kinase domain of the EGFR gene [23-25]. These mutations endow the receptor with increased kinase activity and lead to hyperactivation of downstream signalling cascades. The EGFR active site mutants transform fibroblasts and tracheobronchiolar epithelial cells [26]. Similar findings have been reported for ERBB2 mutations [27] that occur in approximately 2% of NSCLC [28]. Inducible expression of the EGFR active site mutants in transgenic mice has been achieved. Using the tetracycline-inducible system where the transcriptional activator rtTA was expressed under the CC10 promoter [29], conditional expression of EGFR active site mutant proteins in adult lungs led to adenocarcinoma formation [30,31].

**Figure 3:**
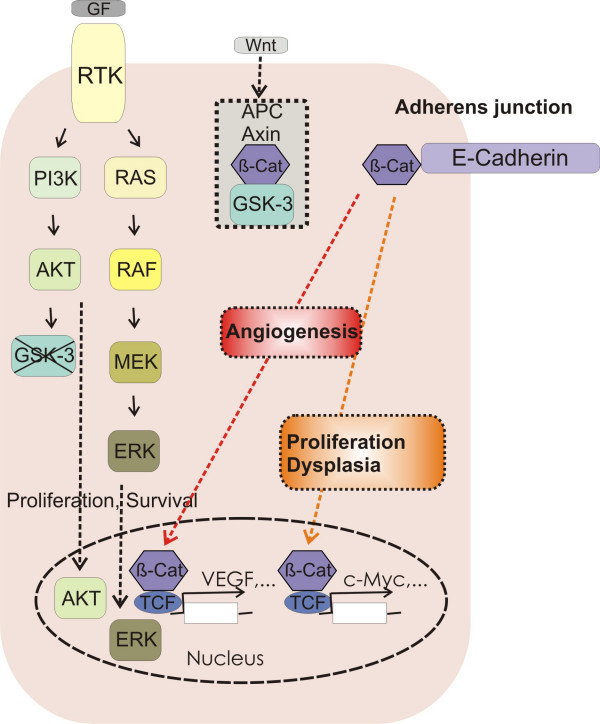
**Regulation of cell adhesion by RAS/MAPK, RAS/PI3K/AKT and β-catenin signalling**. In lung NSCLC, constitutive signalling is caused by mutational activation of components of the RAS/MAPK and/or RAS/PI3K/AKT pathways that endows tumour cells with enhanced proliferation and survival in the absence of a growth factor (GF). Loss of E-cadherin expression and disruption of a functional E-cadherin complex at the adherens junction are two events that occur in human lung cancers. Inducible disruption of the functional E-cadherin complex in a RAF oncogene-driven NSCLC mouse model led to the loss of adherens junction function and deadhesion of neighbouring tumour cells. In addition, nuclear β-catenin activity was instrumental for increased proliferation and survival of tumour cells, as well as the induction of angiogenic factors that facilitated the growth of a tumour vasculature (angiogenic switch) and changes in cell fate integrity (dysplasia).

RAS is a small GTP-binding protein tethered to the inner cell membrane that couples growth factor receptor activation to the downstream RAF-MEK-ERK MAPK cascade (Figure 3). Besides the RAF kinases, several other classes of proteins have been implicated as effectors of RAS [32]. When activated by specific growth factors (for example EGF), the fraction of RAS in the activated RAS-GTP form increases at the expense of the inactive RAS-GDP form. A high proportion of human cancers including those of the lung carry activating point mutations in *RAS *(Figure 2) [33]. These mutations result in the loss of the intrinsic GTPase activity that is needed to return RAS to its inactive GDP-bound state, leaving the mutated RAS permanently active [34]. Several strains of mice with inducible alleles of oncogenic *K-RasG12D *or *K-RasG12V *have been generated [9]. A transcriptional stop element flanked by loxP sites present in the targeted "floxed" allele prevented the expression of the oncogene. The removal of the stop element was achieved either "systemically" (in compound mice harbouring also a Cre recombinase transgene) or through infection of the lungs with recombinant adenovirus expressing Cre recombinase. One of the mouse models allowed for the histochemical visualisation of the cells that expressed the oncogenic *K-RasG12V *allele. Despite widespread lung-targeted expression of RAS oncoprotein upon activation of the oncogenic *K-RasG12V *allele, the majority of the cells expressing the RAS oncoprotein did not gain a proliferative advantage and only few adenomas formed in the lung [35]. Since a substantial number of cells in premalignant tumours underwent oncogene-induced senescence, it has been proposed that this mechanism might limit tumour initiation and/or tumour progression from premalignant adenomas to malignant adenocarcinomas [36]. None of the RAS mouse models showed any evidence for metastatic spread of the tumours. Notably, human lung cancer with *K-RAS *mutations do not appear to progress, either [37].

RAF is a serine-threonine kinase that is activated downstream of RAS [38,39]. RAF activates the kinase MEK (MAP kinase extracellular signal-regulated kinase) which then activates ERK (extracellular signal-regulated kinase, Figure 3). In mammals there exist three structurally similar RAF kinases, A-RAF, B-RAF and C-RAF. Inactivation of the corresponding genes in mice indicates that the three genes fulfil distinct roles [40]. Of the RAF family of protein kinases, B-RAF is the only member to be frequently (~8%) activated by mutation in cancer [41]. Mutations in A-RAF have not been found [42] and mutations in C-RAF are rare in human cancers [43], but C-RAF is overexpressed in ovarian and pulmonary carcinomas [44,45]. The fact that B-RAF can be activated by a single amino acid change, whereas C-RAF and A-RAF require two mutations for oncogenic activation is thought to explain the difference in mutation frequency. Mice expressing in the lung the most common oncogenic B-RAF mutation, B-RAFV600E, have been established [46,47]. One model employed a conditional targeted allele of oncogenic *B-RAFV600E *containing a floxed transcriptional stop element. Lungs infected with an adenovirus expressing Cre recombinase developed benign tumours that grew rapidly for about eight weeks and then showed signs of oncogene-induced senescence (positive staining for p19^ARF ^and Mec1; [46]). The second model used compound mice with switchable (doxycycline-dependent) induction of B-RAFV600E expression controlled by the CC10 promoter. Administration of doxycycline for six weeks led to the formation of adenomas, which regressed upon withdrawal of doxycycline due to tumour cell apoptosis [47].

In another NSCLC model for oncogenic RAF, the SP-C promoter-directed the expression of an oncogenic variant of C-RAF, called C-RAF BXB [48]. The homeodomain containing thyroid transcription factor 1, also called Nkx2.1, controls the SP-C promoter and confers lung-specific gene expression [49]. Importantly, *NKX2.1 *has been found to be amplified in a significant number of lung adenocarcinoma samples [8,50]. Conversely, lungs in *Nkx2.1 *deficient mice have only two main bronchi and instead of an epithelial tree, cystic structures with columnar cells form and surfactant protein genes fail to be expressed [51]. The SP-C promoter is active in the developing lung bud at embryonic day E11 and in the subsequent stages of embryonic lung development and postnatal lung maturation, as well as in alveolar type II pneumocytes of adult lung [52,53]. In the SP-C C-RAF BXB transgenic mouse, lung development proceeds normally despite the fact that the SP-C promoter is active during embryonic development. Lung tumour initiation occurs in postnatal mice at an age of about one week, but only a small fraction of pneumocytes can be transformed. Several hundred adenomas are forming in each lung, consisting of cuboidal, SP-C expressing type II-like cells. Whether the rare adenoma initiation is due to the fact that only a small pool of type II-like cells is susceptible to transformation or results from mosaic C-RAF-BXB expression is currently unclear. Neither impaired alveolar morphology nor function has been noted in the vast majority of pneumocytes. The adenomas grow continuously because a significant fraction of tumour cells is proliferating [54]. The tumour cells did not show overt signs of apoptosis [55,56]. Progression of the adenomas towards adenocarcinomas was only observed upon inactivation of p53 [57], similar to observations made in lung epithelium expressing oncogenic B-RAF [46]. No further progression towards malignancy, such as intravasation into the lymphatic or blood vasculature and outgrowth of organ metastasis has been observed [54], not even after a period of more than 12 months of continuous lung tumour growth [54]. Thus, the adenomas of the SP-C C-RAF BXB model contain proliferating and surviving epithelial tumour cells, making the model suitable to identify tumour progression factors in lung tumourigenesis. Importantly, the adenomas resemble atypical adenomatomous hyperplasia in human lung. Such lesions have been detected incidentally in resected human lung [58] and more recently on a larger scale as a result of computer tomography screening [6]. In sum, mutations along the growth factor receptor kinase-RAS-RAF axis together account for more than 50% of all NSCLC cases. They appear to be functional equivalent as they are nonoverlapping underscoring the important role of this pathway in human lung cancer (Figure 3).

Increased growth factor receptor or RAS signalling not only activates RAF but in addition phosphoinositide-3-kinase (PI3K, Figure 3). PI3K family proteins catalyze the phosphorylation of the 3-position of the inositol ring in phosphoinositide substrates [59]. These phospholipid second messengers recruit the serine/threonine protein kinase B (PKB, also known as AKT) to the cell membrane where its activation by other kinases occurs [60]. Active AKT phosphorylates a variety of proteins that mediate increased cell proliferation and cell survival [60]. Aberrant activation of the PI3K/AKT pathway has been implicated in lung cancer progression [61-63]. Conversely, the PI3K antagonistic enzyme PTEN (phosphatase and tensin homolog) is lost by mutations, deletions or promoter methylation at high frequency in human lung cancer [63-65]. Mice with *K-RasG12D *oncogene activation with a PI3K unable to interact with RAS only formed premalignant lesions but no macroscopic lung tumours [66].

### Breaking the cadherin adhesion complex in RAF-driven adenomas

**Figure 4:**
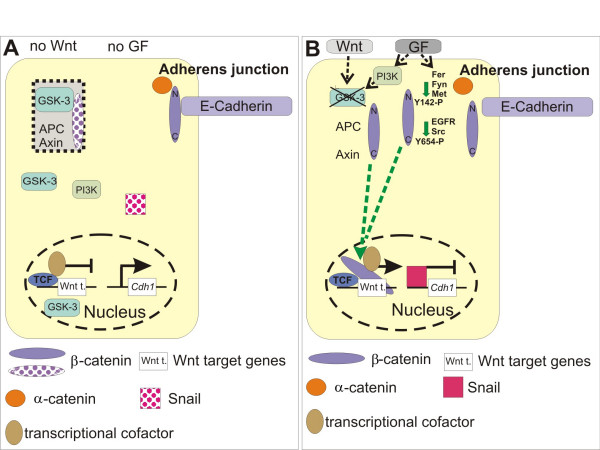
**Regulation of adherens junction and β-catenin stability**. **A**, Localization of β-catenin at the adherens junction occurs in a complex with E-cadherin and α-catenin. β-catenin is also present in the β-catenin-destruction complex where it is being degraded in the absence of Wnt ligand. **B**, Growth factor (GF) as well as oncogene mediated activation of kinases can phosphorylate two conserved tyrosine residues in the N- or C-terminus of β-catenin, leading to a disruption of the adherens complex and formation of a nuclear β-catenin complex. Once in the nucleus, β-catenin can replace co-repressors from TCF/LEF and thereby form activating transcriptional complexes. Activation of Wnt results in the stabilization of β-catenin and its nuclear accumulation where Wnt target genes are turned on (see text for details).

In haematoxylin-eosin stained tissue sections of adenomas from SP-C C-RAF BXB mice, neighbouring tumour cells seemed to be in direct contact and therefore probably maintained the expression of adhesion molecules. Immunostaining revealed that E-cadherin was indeed expressed uniformly in the adenomas of SP-C C-RAF BXB mice [54]. E-cadherin is the dominant cadherin in epithelial tissues [67]. The extracellular part of E-cadherin forms calcium-dependent homophilic dimers thus mediating cell-to-cell adhesion and joining neighbouring epithelial cells. The cytoplasmic domain of E-cadherin is organized in a heteromeric complex with β-catenin bound directly to a conserved sequence in the distal part of its cytoplasmic domain and α-catenin, a relative of vinculin, bound to the N-terminus of β-catenin. E-cadherin and β-catenin are phosphoproteins and phosphorylation acts as a biochemical switch to regulate complex formation at adherens junctions and shuttling between subcellular destinations (see below). Indeed, mutational analysis of a serine-rich catenin-binding domain of cadherin revealed that serine phosphorylation is important for E-cadherin-catenin interaction [68]. It appears that soon after translation, β-catenin – α-catenin dimers form a complex with cadherin before being transported to the cell surface [69]. This core complex at the adherens junction (Figure 4) forms a dynamic link to the actin cytoskeleton [70,71]. Other molecules have also been found to become associated with the core complex. These include p120-catenin, which interacts directly with the membrane-proximal region of the cadherin cytoplasmic domain and may assemble non-receptor tyrosine kinases such as Fyn, Yes and Fer to the complex [72,73]. In addition, the tyrosine phosphatase PTP1B may be recruited to p120-catenin, at least in fibroblasts and neural cells which express N-cadherin instead of E-cadherin [74].

β-catenin has a second life in the canonical Wnt (wingless/int) cascade [75]. In the absence of a Wnt ligand, it is present in the "β-catenin-destruction complex", an assembly of proteins that includes the scaffolding protein Axin, adenomatous polyposis coli (APC) protein, casein kinase Iα and glycogen synthase kinase-3 (GSK-3, [76]). GSK-3 phosphorylates β-catenin at serine/threonine residues N-terminal to the first armadillo repeat (Figure 4A, and see below). The phosphorylated motif allows the binding of a component of the E3 ubiquitin ligase, followed by ubiquitination and degradation by the proteasome. When Wnt signalling becomes activated, β-catenin escapes the degradation complex and migrates to the cell nucleus (Figure 4B). Since β-catenin does not bind to DNA itself, it has to form a complex with TCF/LEF (T-cell factor/lymphoid enhancer factor) transcription factors [77]. These complexes assemble components of the general transcription machinery and proteins involved in chromatin modification to induce the expression of a specific subset of gene targets which mainly determine cell proliferation and cell fate [78]. These include cyclin D1 and c-Myc which are up-regulated in colon cancers [78]. c-Myc is a key mediator of activated Wnt signalling in murine intestinal cancer [79]. GSK-3 family proteins (GSK-3α and GSK-3β) are constitutively active in resting cells. The transcription factor Snail has been identified as one target of GSK-3 leading to the nuclear export and destruction of Snail by the proteasome [80]. GSK-3 activity is suppressed in response to external signals and involves AKT-mediated phosphorylation at an N-terminal motif [81]. Inactivation of GSK-3β by phosphorylation of the N-terminal serine might be involved in tumour progression because it leads to increased levels of c-Myc (cellular Myelocytomatosis, also called MYC, encoded by the *MYC *gene, [82]; see below). Knock-in mice where the N-terminally serines of GSK3 proteins were replaced by alanines had no defects in Wnt signalling indicating that GSK-3 activity in the Wnt pathway is regulated in a manner distinct from phosphorylation of the N-terminal serine that may involve disassembly of the β-catenin-destruction complex [83,84]. Another consequence of GSK-3 inactivation is that Snail will accumulate in the nucleus and repress E-cadherin expression (Figure 4B).

We addressed the functional consequences of weakening or destroying cell-to-cell adhesion in the adenomas of SP-C C-RAF BXB mice using two different genetic approaches, conditional ablation of the *Cdh1 *gene in mice with a "floxed" *Cdh1 *gene [85] or switchable expression of dominant negative E-cadherin. As expected, the ablation of functional E-cadherin caused deadhesion among tumour cells [54]. In addition, in a fraction of tumour cells, nuclear β-catenin was observed, indicating that upon removal of E-cadherin, β-catenin was not completely destroyed but transported to the nucleus. Thus, the release of β-catenin from the cadherin complex not only affected the integrity and function of the adhesion complex, but may also have made it available for signalling in the nucleus. This observation is in line with a previous investigation in non-timorous cells, where the formation of a transcriptional β-catenin complex has been observed upon loss of E-cadherin [86].

Increased tumour cell proliferation was observed in vivo, in line with the up-regulation of cyclin D1 and c-Myc in tumour cells 36 hours after induction of dominant negative E-cadherin [54]. After induction of dominant negative E-cadherin or inactivation of the *Cdh1 *gene, massive formation of blood and lymph vessels, termed the "angiogenic switch" [87] was observed in the adenomas [54]. Vascular endothelial growth factors A and C were identified as β-catenin target genes in vivo and in NSCLC tumour cells [54]. The finding that vessel induction by dominant negative E-cadherin was reversible in the early phase of induction, but was no longer reversible after long-term ablation of E-cadherin may be explained by the eventual acquisition of a tumour vessel signature [88]. Vascularized tumours developed invasive fronts and gave rise to micrometastasis in thoracic lymph nodes and bone marrow. It is notable that the presence of micrometastases in lymph nodes in lung adenocarcinoma patients correlates with poorer outcomes as compared to those lacking detectable micrometastases [89]. Progression towards malignancy upon ablation of E-cadherin in epithelial tumour cells was not only observed in RAF-driven lung tumourigenesis [54] but also in a mouse breast cancer model with epithelium-specific inactivation of p53 [90]. Combined loss of E-cadherin and p53 led to accelerated development of invasive and metastatic mammary carcinomas [90].

### Many roads to nuclear β-catenin signalling in cancer

Frequent mutational activation of Wnt/β-catenin signalling has been observed in various types of human cancers [91]. With respect to genes specifying the adhesion complex, mutations in E-cadherin (*CDH1*) have been found in gastric and colon cancer but have not yet been reported in lung cancer. The most frequent colorectal cancer-associated genetic lesions are loss-of-function mutations in the *APC *gene and they result in sustained β-catenin signalling [91]. Activating mutations have also been identified in the *CTNNB1 *gene, which encodes β-catenin, in colon cancer cells [92] as well as in melanoma cell lines [93]. In addition, deletions in exon 3 of *CTNNB1 *have been detected in colorectal carcinomas and transcripts lacking exon 3 are translated into a stabilized β-catenin with dominant nuclear β-catenin signalling [94,95]. Colon-restricted conditional expression of stabilized β-catenin mutant in transgenic mice led to the formation of adenomatous intestinal polyps and colon microadenomas [96]. In human lung cancer, mutations have not been reported for *APC *and are rare in *CTNNB1 *(3%) [97]. Activation of Wnt signalling upstream of β-catenin has been observed in a low number of lung cancer samples [98]. Whether nuclear expression of β-catenin correlates with poor or better prognosis in NSCLC is unclear [98].

I will now discuss alternative mechanisms that may result in nuclear β-catenin signalling in cancer cells in the absence of overexpressed Wnt ligands or mutational activation in the canonical Wnt/β-catenin signalling cascade. Repression of *CDH1 *expression by epigenetic mechanisms might lead to a loss of E-cadherin function in the absence of *CDH1 *loss-of-function mutations. Aberrant methylation of normally unmethylated CpG islands in the promoter regions of genes has been correlated with transcriptional inactivation of several genes in human cancer. Silencing of E-cadherin expression by promoter CpG methylation was found in a variety of human epithelial carcinoma cell lines [99]. In primary NSCLC cancers CpG island methylation was detected at a frequency of 18% with methylation-specific PCR [100]. Secondly, transcription factor-mediated suppression of E-cadherin expression might be at play. Indeed, Twist, a transcription factor that suppresses E-cadherin expression and cell-to-cell adhesion, has been found to be up-regulated in breast cancer [101]. The contribution of Twist towards malignancy may use as an additional mechanism the induction of miR-10b in breast cancer cells [102]. miR-10b belongs to the class of regulatory small cellular RNAs, termed microRNA (miRNA), that act as mediators of the RNA interference pathway. Twist-induced miR-10b, and miR-10b was found to inhibit the expression of the HOXD10 protein, permitting the expression of the pro-metastatic gene product, RHOC [102].

As a further possibility we have to consider that β-catenin may be released by phosphorylation from complexes at the membrane and subsequently enter the nuclear signalling pool. The three-dimensional structure of β-catenin and the structure of the cadherin/β-catenin complex have been elucidated [103,104]. β-catenin has 12 armadillo repeats; each repeat is composed of three α-helices. The binding interface of β-catenin with E-cadherin involves armadillo repeats 5–12 whereas the interaction of β-catenin with the DNA binding factor TCF-4 uses armadillo repeats 3–8. Therefore, some flanking armadillo repeats of β-catenin would be suitable for posttranslational modifications that may disrupt specifically E-cadherin or α-catenin binding without ablating the interaction with TCF. As detailed below, phosphorylation of β-catenin at two critical tyrosine residues, tyrosine-142 and tyrosine-654, has been shown to disrupt the E-cadherin/β-catenin adhesion complex with concomitant β-catenin nuclear activity (Figure 4). Since both modifications lie outside the interaction domain with LEF/TCFs, complex stability of β-catenin with LEF/TCFs would not be affected by phosphorylation.

Cell surface transmembrane tyrosine kinase receptors modulate the adhesion complex by phosphorylation, as demonstrated for the EGFR [105]. This work showed that the EGFR is associated with the E-cadherin-β-catenin adhesion complex and that the receptor can phosphorylate β-catenin in vitro and in three epithelial cell lines. Furthermore, an association of β-catenin with receptor ERBB2 together with phosphorylation of β-catenin has been shown in gastric cancer cell lines [106-108]. Activated RAS has been shown to induce tyrosine phosphorylation of β-catenin leading to disruption of adherens junctions in an epithelial cell line [109]. In transgenic mice, intestinal expression of an oncogenic *K-RasG12V *allele in conjunction with a loss-of-function allele of APC was shown to increase nuclear β-catenin signalling [110]. This combination of genes led to increased intestinal tumour initiation and accelerated tumour progression [110]. Activation of the tyrosine kinase Src in epithelial MDCK (Madin-Darby canine kidney) cells impaired cell adherens junction function via phosphorylation of β-catenin [111]. Src has been shown to phosphorylate β-catenin on tyrosine-654 in vitro and evidence was provided that this phosphorylation is important for the disruption of the E-cadherin/β-catenin complex in a colorectal cell line [112]. Furthermore, tyrosine-654 phosphorylation in vitro induced the formation of a β-catenin complex with the nuclear TATA binding protein [113]. Using a Src kinase inhibitor, tyrosine phosphorylation of β-catenin was blocked and expression of β-catenin target genes was reduced in a colorectal cancer cell line [114]. In addition, cell-to-cell adhesion was restored in the presence of the Src kinase inhibitor, whereas cell migration was strongly reduced [114]. In fibroblast cells, overexpression of another tyrosine kinase, Fer led to a disruption of adherens junction complexes [115].

Tyrosine-142 is present in the first armadillo repeat which overlaps with the α-catenin binding region of β-catenin. Phosphorylation of this residue induced the dissociation of α-catenin from the E-cadherin/β-catenin complex with concomitant loss of cell aggregation [116]. Tyrosine-142 is phosphorylated by Fyn, Fer and Met, the tyrosine kinase cell surface receptor for hepatocyte growth factor (HGF). Stimulation of the Met receptor in conjunction with overexpression of Bcl 9-2, an adaptor protein that links β-catenin to transcriptional coactivators, in epithelial MDCK cells did not only disrupt cell-to-cell adhesion but pulled β-catenin from an adhesion complex into a nuclear complex with Bcl 9-2. Thus, phosphorylation of tyrosine-142 weakened α-catenin binding to β-catenin in favour of a nuclear β-catenin complex with Bcl 9-2 [117]. In primary hepatocytes, where a significant fraction of β-catenin is bound to Met, HGF induced nuclear translocation of β-catenin [118]. We also have to consider that trimeric Met/E-cadherin/β-catenin complexes may exist in cancer cells [119,120] and that Met activation may occur in a significant fraction of lung adenocarcinoma samples [121]. In NSCLC, intronic mutations occur in *MET *[122]. They lead to the expression of a receptor with a deleted juxtamembrane domain with sustained kinase activity after HGF stimulation [122]. Another possibility that might switch β-catenin from an adhesive into a signalling factor is a conformational change in the C-terminus of β-catenin [123]. In sum it is clear that there are additional routes to nuclear β-catenin signalling, parallel to the Wnt pathway. They operate by weakening the association of E-cadherin with β-catenin in the adhesion complex, using a variety of mechanisms and thus make β-catenin available as a nuclear signalling partner in cancer cells, including those present in lung adenocarcinoma. Further studies are required to define at what stage of tumourigenesis any of the proposed mechanisms are relevant.

### β-catenin signalling and the induction of dysplasia

Why did the disseminated tumour cells forming a micrometastasis in compound mice with oncogenic RAF and disrupted adherens junctions fail to colonize and grow into a macrometastasis? One obvious explanation may be the failure of the micrometastasis to acquire a "metastasis virulence gene" that would allow for colonization in the new tissue [124]. A second possibility comes from observations in several human cancers showing that loss of E-cadherin function is accompanied by the expression of other cadherin family members, termed "cadherin switch" [125]. According to this model, tumour cells forming a micrometastasis have to re-establish adhesion at the new organ site in order to grow. The conditional transient re-expression of E-cadherin at the stage of micrometastasis should allow testing this hypothesis. A third scenario may be inappropriate β-catenin activity. When systemic, long-term activation of β-catenin signalling was induced in six week-old SP-C C-RAF BXB mice with lung adenomas via the expression of dominant negative E-cadherin, RT-PCR analysis of tumour cell mRNA revealed the expression of genes (*Cdx1, Atoh1*) that are normally expressed in the intestine indicating that an epigenetically-driven change in cell fate had occurred [54]. As it is not clear, whether the lung cells changed morphologically into intestine cells or acquired a broader range of pluripotency or self renewal activity, I shall call this form of epigenetic plasticity dysplasia [126]. During embryonic and postnatal organ development and cell differentiation, cell fate programmes are altered in response to extracellular cues. Under physiologic conditions, these cues (type and dose of signalling molecules) are provided by neighbouring cells or through the circulation. The cues manifest in the epigenetic regulation of gene expression by heritable modification of the DNA (principally methylation of CpG dinucleotides) and through altering chromatin. Histone proteins function as building blocks that package the DNA into nucleosomes and distinct histone modifications form a "histone code" [127]. Modifications that condense chromatin, modify the access of transcription factors to DNA or recruit other complexes such as the Polycomb complex to chromatin, occur during development as well as in stem cells and cancer [128,129]. The modifications serve as cell fate-specific marks. They are generally passed on unchanged from one cell to its daughter cells and secure the faithful expression of differentiation-associated genes.

Therefore, it appeared that there may be no way back for a differentiated committed cell to an earlier fate after it has lost its pluripotent capacity. However, there are examples for transdifferentiation, the transformation of one differentiated cell into another [126]. Furthermore, epigenetic changes are reversible and have been experimentally induced by somatic cell nuclear transfer and cell fusion, and more recently via combined expression of four specific transcription factors, also termed "magic brew" [130]. The four factors (Oct3/4, Sox2, Klf4 and c-Myc, with c-Myc being an oncogene) reprogrammed mouse fibroblasts into induced pluripotent stem (iPS) cells that resemble embryonic stem cells in terms of morphology [131]. The achievement of bona fide reprogramming towards pluripotency was proven, since the iPS cells, after injection into early embryos, differentiated into all normal adult cell types [132-134]. While the first report [131] used drug selectable markers driven by promoters that are active in embryonic stem cells for the isolation of rare iPS cells, morphology alone can also be used to detect iPS clones [135]. It also appears that two of the factors, Myc and Klf-4 increase the frequency of reprogramming and are not strictly necessary [135,136].

The *MYC *proto-oncogenes are essential activators of cell proliferation and have emerged as some of the genes most commonly deregulated in cancer [137]. Activation of *MYC *genes in human cancer occurs by amplification or loss of transcriptional control, which results in MYC protein overexpression. In NSCLC, *MYC *gene copy number is found to be amplified in a fraction of specimen [8]. The up-regulation of c-Myc at the post-transcriptional level also appears to be of instrumental importance for c-Myc to promote tumour progression [138]. The half-life of c-Myc is controlled by a number of sequential, reversible phosphorylations on two conserved residues, serine-58 and serine-62. In response to MAPK signalling, c-Myc is phosphorylated on serine-62; parallel activation of PI3K/AKT leads to the inactivation of GSK3, with threonine-58 remaining unphosphorylated [82]. Active GSK-3 however can phosphorylate c-Myc on threonine-58 if serine-62 is phosphorylated which subsequently may lead to its degradation through the activity and co-operation of other enzymes such as prolyl isomerase 1 and protein phosphatase 2A [138].

In transgenic mice expressing Nmyc from the SP-C promoter progenitor cells in the developing distal lung expanded in numbers and their differentiation was blocked leading to disturbed lung function and perinatal death [53]. SP-C promoter-directed overexpression of c-Myc in transgenic mice did not disturb lung development and induced tumour formation in the lung only after long latency [139], suggesting that a second hit, presumably the mutational activation of an oncogene, may be needed for tumour initiation. In contrast, conditional Myc overexpression in other tissues such as the haematopoietic system or the liver induced carcinomas with short latencies [140,141]. Importantly, abrogation of Myc expression caused tumour regression arguing against additional mutational events during carcinoma formation. When hepatocellular carcinoma cells or distant lung metastases derived from the carcinomas were transplanted under the skin of SCID mice, tumours engrafted in the recipient animals. The tumours regressed after abrogation of Myc expression and the residual scar tissue under the skin contained differentiated cells resembling hepatic lobules [141], indicating that the transplanted metastatic cells could regain their differentiated phenotype.

According to a classical model, c-Myc is a basic-helix-loop-helix zipper transcription factor that dimerizes with Max leading to the activation of genes that contain binding sites for Myc-Max complexes. The identification of thousands of putative c-Myc target genes [142], the finding that about half of all DNA sites binding c-Myc were intergenic (>10 kb away from transcriptional start sites [143], and the finding that c-Myc promotes DNA replication through a non-transcriptional mechanism with subsequent DNA damage [144] indicate that the classical model does not fully explain tumourigenic c-Myc function. Conditional disruption of N-Myc in distal lung epithelial cells inhibited distal lung proliferation and induced premature differentiation indicating that it is required for maintaining a distal population of undifferentiated, proliferating progenitor cells [53].

A form of epigenetic plasticity similar to that noted in adult mice with oncogenic RAF-driven lung tumours with disruption of adherens junctions and nuclear β-catenin signalling [54] had been observed before in embryonic lung epithelium in transgenic mice with SP-C promoter-directed expression of a constitutively active fusion protein of β-catenin and the transcription factor Lef1 [145]. These lungs lacked differentiated lung cell types and showed a hyperproliferative epithelium expressing *Cdx1, Atoh1 *and other genes normally expressed in intestinal epithelium. Another example of reprogramming has been observed upon introduction of activated β-catenin into either the common lymphoid or common myeloid progenitors [146]. Multipotential cells giving rise to both lymphoid and myeloid offspring were generated but apparently only single-step reversion in the developmental hierarchy had occurred, because upon grafting into irradiated mice, no full reconstitution was observed [146]. Therefore, the question arises whether step-wise reversion in epigenetically controlled developmental programmes of gene expression may contribute to malignancy. Indeed, it has been proposed, that tumour-restricted "recapitulation" of signatures that are normally restricted to embryonic development might contribute to neoplastic progression [147,148]. In line with this hypothesis, Wnt signalling has been proposed to increase the self-renewal capacity of haematopoietic stem cells [149]. However, two groups have recently reported [150,151] that inducible overexpression of a stabilized β-catenin increased the proliferation of haematopoietic cells as expected but led to an exhaustion of functional haematopoietic stem cell potency. Mechanistically, the loss of a haematopoietic stem cell signature (HoxB4, Pu.1, Bmi-1 expression) was observed. Whether a similar stem cell-suppressive action is at play in lung tumours with oncogenic C-RAF and dominant-negative E-cadherin expression has to be determined.

## Conclusion

Two recent studies have evaluated the consequences of the loss of E-cadherin in conjunction with either oncogenic RAF kinase expression or loss of p53 for malignant progression of epithelial tumours. The investigation of the breast cancer model revealed that combined loss of E-cadherin and p53 in mammary epithelial cells induced metastastic carcinoma that resembles invasive lobular carcinoma. In the lung NSCLC model, disruption of E-cadherin led to vascularized tumours that grew rapidly and gave rise to micrometastasis. Nuclear β-catenin signalling was instrumental for up-regulation of vascular endothelial growth factor expression and epigenetic plasticity. Published data are being reviewed on how receptor and non-receptor kinases impinge on the stability of the adherens junction complex. Evidence suggests that kinases may modulate nuclear β-catenin signalling. In a cooperative manner, kinases and nuclear β-catenin may be instrumental for the expression of gene targets in the lung which in turn is involved in malignant progression. I finally discuss what mechanisms may block the step from micrometastasis to macrometastasis and how E-cadherin function, together with oncogenic and nuclear β-catenin signalling might control this important step of malignancy.

## List of abbreviations

APC: adenomatous polyposis coli; BADJ: bronchio-alveolar duct junction; BASC: bronchio-alveolar stem cell; CC: Clara cell; c-Myc: cellular Myelocytomatosis; EGFR: epidermal growth factor receptor; ERK: extracellular signal-regulated kinase; HGF: hepatocyte growth factor; LEF: lymphoid enhancer factor; MAPK: mitogen-activated protein kinase; MEK: MAP kinase extracellular signal-regulated kinase; NSCLC: non-small-cell lung cancer; PCR: polymerase chain reaction; PI3K: phosphoinositide-3-kinase; RT-PCR: reverse transcription- polymerase chain reaction; rtTA: reverse transcriptional transactivator; SCID: severe combined immune deficiency; SP-C: surfactant protein-C; Sca1: stem cell antigen 1; TCF: T-cell factor; VEGF: vascular endothelial growth factor; Wnt: wingless/int.

## Competing interests

The author declares that they have no competing interests.
